# Antimicrobial stewardship and quality improvement strategies in critical access hospitals: a process evaluation of an intensive quality improvement cohort

**DOI:** 10.1017/ash.2024.458

**Published:** 2024-11-11

**Authors:** Zahra Kassamali-Escobar, Whitney Hartlage, Jeannie D. Chan, Alyssa Castillo, Natalia Martinez-Paz, Maria Bajenov, Rupali Jain, John B. Lynch, Chloe Bryson-Cahn

**Affiliations:** 1 Center for Stewardship in Medicine, University of Washington School of Medicine, Seattle, WA, USA; 2 School of Pharmacy, University of Washington, Seattle, WA, USA; 3 Fred Hutchinson Cancer Center, Seattle, WA, USA; 4 Veteran’s Affairs Salt Lake City Health Care System, Salt Lake City, UT, USA; 5 Division of Allergy and Infectious Diseases, Department of Medicine, University of Washington School of Medicine, Seattle, WA, USA; 6 Division of Infectious Diseases, Department of Medicine, University of Colorado School of Medicine, Aurora, CO, USA; 7 International Training and Education Center for Health (I-TECH), Department of Global Health, University of Washington, Seattle, WA, USA

## Abstract

We describe our experience implementing an intensive quality improvement cohort pilot focused on managing asymptomatic bacteriuria in 19 critical access hospitals. Participation in the pilot was high, and almost all sites identified an improvement goal and collected clinical data. Barriers to implementation included staffing shortages, turnover, and lack of bandwidth.

## Introduction

Rural healthcare facilities and critical access hospitals (CAHs) serve approximately 20% of the United States population.^1^ CAHs have ≤ 25 licensed beds and are located at least 35 miles apart.^2^ Although not all rural hospitals are CAHs, they face similar geographic barriers and serve similar patient populations.^3,4^ The University of Washington Center for Stewardship in Medicine (UW CSiM) connects UW clinical specialists in infectious diseases with diverse staff from 82 rural and CAHs across 9 states via a tele-antimicrobial stewardship program, UW-TASP ECHO.^5^ In 2021, in response to a new federal requirement for the state Office of Rural Health (ORH) Flex programs to build quality improvement (QI) projects, UW CSiM began an intensive quality improvement cohort (IQIC) pilot focused on stewardship of asymptomatic bacteriuria (ASB), the presence of bacteria in the urine without signs and symptoms of a urinary tract infection (UTI). We selected ASB because we previously demonstrated a 50% prevalence of ASB among urine cultures collected in CAHs—of which 90% were inappropriately treated with antibiotics.^7,8^ Here, we assess the level of engagement during IQIC, report on barriers to implementation and describe experiences overall with the program.

## Methods

The IQIC pilot was developed over 6 months by UW CSiM faculty with investment and input from ORH Flex programs in Arizona, Idaho, Oregon, Utah, and Washington.

### Program format

This year-long program operated from September 2021–August 2022, offered eight educational sessions delivered monthly by UW ID pharmacists and physicians over Zoom, quarterly mentoring sessions with IQIC faculty, and provided the tools for CAH personnel to submit de-identified data on ASB prescribing practices at their site. The UW CSiM website housed an online dashboard for each site to document progress toward a QI goal using a plan-do-study-act framework. The website also included links to treatment guidelines and educational handouts targeting both providers and patients; many were modified with permission from resources available freely from the Massachusetts Coalition for the Prevention of Medical Errors.^9^

### Implementation

State ORH Flex programs recruited CAHs already participating in TASP ECHO for the IQIC pilot. The monthly didactic curriculum focused on QI methods and appropriate diagnosis and management of UTIs. In one-on-one mentoring sessions, sites discussed stewardship plans and any barriers encountered. During the second half of the year, sites focused on data collection using a REDCap data form,^10^ and process mapping to set a QI goal.

### Data collection

We defined four process measures for participating CAHs: (1) create and implement a plan-do-study-act cycle (PDSA) related to ASB, (2) attend at least 6 of 8 education sessions in real time, (3) attend at least 3 of 4 one-on-one meetings, and (4) collect local data to identify ASB rates and rates of inappropriate treatment, with no minimum number of cases set. We surveyed participants for their self-reported knowledge of ASB before and after joining the program using a Likert scale from 1 (beginner), to 5 (expert). Barriers to implementation were discussed and recorded by one investigator (ZKE) during one-on-one meetings with participating sites. At completion of the cohort, notes were reviewed, and barriers were categorized by “knowledge and attitude,” “organizational,” and “bandwidth”.^10^ Because data collection was frequently reported as a barrier to implementation, a “data” category was added. Data were analyzed using descriptive statistics. An analysis of the quantitative data collected during this pilot has been published elsewhere.^10^

## Results

Thirty-two individuals from 19 CAHs in five states participated in the yearlong IQIC pilot: 3 hospitals were from Washington, 5 from Oregon, 6 from Idaho, 2 from Utah, and 3 from Arizona. The professional training of the 32 individuals participating was: 17 pharmacists, 13 infection preventionists, 1 laboratory personnel, and 1 physician. Eighteen of 19 hospitals (95%) set a goal and documented their PDSA cycle on the online dashboard. Full implementation of a PDSA cycle was not completed by any participating CAH within the 1-year time frame. Participants from 16 of 19 CAHs (84%) attended at least 6 monthly education sessions in real-time. The median number of one-on-one quarterly sessions attended per site was 3 (range, 1–4). Seventeen hospitals (89%) collected local data (Table 1). The median number of cases submitted per site was 45 [IQR 34–90].

**Table 1.** Engagement of 19 CAHs in the UW CSiM 12-month Intensive Quality Improvement Cohort (IQIC), 2021*–*2022

Engagement Indicator	Measurement	Results
-Attended monthly education sessions	Number of education sessions attended in real-time (out of 8 possible)	Median 8, IQR 2–8
-Attended quarterly one-on-one mentoring sessions	Number of mentoring sessions attended (out of 4 possible)	Median 3, IQR 2.75–4
-Set an improvement goal	Goal documented in online dashboard	N = 18 (95%)
-Collected local data on topic	Submitted any case data via online data collection form	N = 17 (89%)

IQR, interquartile range.

Fifteen participating CAHs (79%) responded to the survey. Seven respondents (47%) self-described their stewardship team’s knowledge of ASB as beginner (score = 1), 4 (27%) as advanced beginner (score = 2), and 4 (27%) as competent (score = 3) prior to joining IQIC. None described themselves as proficient (score = 4) or expert (score = 5). After participating in IQIC, 3 (20%) described their team’s knowledge of ASB as advanced beginner, 6 (40%) as competent, and 6 (40%) as proficient. None described themselves as expert or beginner.

The two most common self-reported barriers to implementing IQIC activities included bandwidth due to heavy workloads, largely due to having multiple roles within their facilities (for example: serving as antimicrobial stewardship leader plus pharmacy director) and difficulty obtaining data (often due to lack of information technology support to extract data). Knowledge and attitude barriers included perceived resistance to practice change among personnel and clinician lack of awareness of the most recent clinical guidelines for UTI. Organizational barriers included high staff turnover, limited facetime with staff, and use of locum or traveling clinicians who are often less familiar with institutional practices and guidelines. Data barriers included low frequency of urine cultures, and long turnaround time on urine cultures (Table 2).

**Table 2.** Barriers to implementation, and most frequently reported type of barrier for each category, reported by 19 CAHs in the UW CSiM 12-month intensive quality improvement cohort (IQIC), 2021–2022

Knowledge and Attitude n = 6	Organizational n = 13	Bandwidth n = 9	Data n = 9
Provider resistance	Staff turnover	Data submissions are time consuming	Difficulty getting data
RN resistance	Staff shortage	No time to participate	Poor quality data from EMR
Hospital staff do not use order sets	Need to get buy-in from central ASP	No bandwidth	Inability to change UA reflex criteria or process
Providers do not support ASP	Lack of ED contact person	Competing priorities at hospital	Long TAT for urine culture
Lack of awareness of current guidelines for UTI and ASB	Hand off between staff	No time to perform data collection	Low frequency of urine culture
	Limited facetime with staff		Culture results come as a.pdf file
	Educating weekend, locum and night staff		Lack of symptom data in charts
	Lack of consistency with personnel		Reports not run in timely manner

RN, registered nurse; ED, emergency department; ASP, antimicrobial stewardship program; EMR, electronic medical record; TAT, turnaround time; UA, urinalysis; UTI, urinary tract infection; ASB, asymptomatic bacteriuria; CAH, critical access hospital.

## Discussion

Overall engagement of CAHs in the IQIC pilot program was high, as measured by attendance, setting improvement goals, and collecting local data. This indicates interest and opportunity to increase antimicrobial stewardship support to CAHs in rural communities. High participation in one-on-one mentorship sessions likely reflects the value placed on personalized support. Barriers to implementation resemble those previously published in the literature: limited time and bandwidth to implement QI, problems with staff shortages, and staff who are resistant to change.^11^ In this pilot, an additional barrier identified was difficulty in obtaining basic data on urine testing, symptoms and treatment rates—which highlights a key issue to address in future support of antimicrobial stewardship work in CAHs.

While sites self-reported an improvement in knowledge, we acknowledge a survey is a limited tool to measure this change. Pharmacists and infection preventionists were the two most common backgrounds of participants in the cohort. Those who had built rapport either over time or through participating in this quality improvement cohort with their hospital staff demonstrated the greatest capacity for program-building. This was counterbalanced with multiple competing responsibilities including management of COVID-19 (cohort participation occurred in 2021), and updating/validating electronic medical record systems and other hospital technologies.

During the pilot, we noted that while CAHs have common characteristics, like remoteness and resource scarcity, the processes and individuals who determine outcomes at individual CAHs are unique. This underscores that a “one-size-fits all” approach to stewardship of ASB at CAHs does not guarantee success. Successful development of realistic and achievable goals is a skill that often requires pre-work, like stakeholder engagement, process mapping, and data collection to understand the current state. In some CAHs, the entire IQIC year was needed just to obtain data to inform the baseline state. For these reasons, outcomes evaluation of programs like IQIC may be difficult in the short term (≤1 year), and we found it most informative to focus on process evaluation through tracking of site participation and site feedback. Staff turnover, overwork, and burnout were salient issues during the IQIC pilot; in a CAH, this is even more challenging due to their smaller size but not smaller scope. Despite the challenges, the majority of CAHs continued to consistently engage with IQIC, including committing to their projects for 1 to 2 more years through subsequent IQIC cohorts [Ciarkowski et al, pending publication]. This speaks to the interpersonal connections and relationships forged through the IQIC pilot and the power of connecting CAHs together. This 1-year pilot program has been expanded into a two-year curriculum and repeated with an additional 20 rural and critical access hospitals. We believe a combination of quality improvement tools, syndromic education, and accountability and mentorship can serve as an effective means for quality improvement in CAHs.

## Supporting information

Kassamali-Escobar et al. supplementary material Kassamali-Escobar et al. supplementary material

## Supplementary material

For supplementary material accompanying this paper visit http://doi.org/10.1017/ash.2024.458. click here to view supplementary material
