# Supplementary material for: Antimicrobial stewardship and quality improvement strategies in critical access hospitals: a process evaluation of an intensive quality improvement cohort
Source: Antimicrob Steward Healthc Epidemiol. 2024 Nov 11;4(1):e201. doi: 10.1017/ash.2024.458 (PMC11574594; doi:10.1017/ash.2024.458)
Supplement: Kassamali-Escobar et al. supplementary material [file S2732494X24004583sup001.docx]

**IQIC ASB 101 Syllabus**

September 2021- August 2022

| **Date** | **Educational Session** |
| --- | --- |
| September | Introduction to QI and application to ASB Behavior management |
| October | Deeper dive into ASB Cases  SMART Goals |
| November | Process Mapping  Antibiotic Harms |
| December | Behavior Change and Productive Conversations  Flexing and Reflexing: A Case in Urine Culture Management |
| January | Reflecting on Reflexing: A Pro/Con Debate |
| March | Project Management Fundamentals and Tools for Success  PDSA Cycles |
| April | Telling Your Story: Framing and Narrative |
| June | ASB Findings – Summary of the data collected |
| July | Final presentations for each of the sites (5 min) |
| August | Final Presentations for each of the sites (5 min) |

Abbreviations: ASB- asymptomatic bacteriuria; IQIC- intensive quality improvement cohort; min- minute; PDSA- plan, do, study, act; Q&A- question and answers; QI- quality improvement; SMART- specific, measurable, achievable, relevant, time-bound
